# Trends in Poor Health Indicators Among Black and Hispanic Middle-aged and Older Adults in the United States, 1999-2018

**DOI:** 10.1001/jamanetworkopen.2020.25134

**Published:** 2020-11-11

**Authors:** Michelle Odlum, Nathalie Moise, Ian M. Kronish, Peter Broadwell, Carmela Alcántara, Nicole J. Davis, Ying Kuen K. Cheung, Adler Perotte, Sunmoo Yoon

**Affiliations:** 1 Columbia University Irving Medical Center, New York, New York; 2 Division of General Medicine, Department of Medicine, Columbia University Irving Medical Center, New York, New York; 3 Center for Interdisciplinary Digital Research, Stanford University, Stanford, California; 4 Columbia University School of Social Work, New York, New York; 5 Clemson University School of Nursing, Clemson, South Carolina; 6 Department of Biostatistics, Columbia University Irving Medical Center, New York, New York; 7 Department of Biomedical Informatics, Columbia University Irving Medical Center, New York, New York; 8 Data Science Institute, Columbia University, New York, New York

## Abstract

**Importance:**

Adults who belong to racial/ethnic minority groups are more likely than White adults to receive a diagnosis of chronic disease in the United States.

**Objective:**

To evaluate which health indicators have improved or become worse among Black and Hispanic middle-aged and older adults since the Minority Health and Health Disparities Research and Education Act of 2000.

**Design, Setting, and Participants:**

In this repeated cross-sectional study, a total of 4 856 326 records were extracted from the Behavioral Risk Factor Surveillance System from January 1999 through December 2018 of persons who self-identified as Black (non-Hispanic), Hispanic (non-White), or White and who were 45 years or older.

**Exposure:**

The 1999 legislation to reduce racial/ethnic health disparities.

**Main Outcomes and Measures:**

Poor health indicators and disparities including major chronic diseases, physical inactivity, uninsured status, and overall poor health.

**Results:**

Among the 4 856 326 participants (2 958 041 [60.9%] women; mean [SD] age, 60.4 [11.8] years), Black adults showed an overall decrease indicating improvement in uninsured status (β = −0.40%; *P* < .001) and physical inactivity (β = −0.29%; *P* < .001), while they showed an overall increase indicating deterioration in hypertension (β = 0.88%; *P* < .001), diabetes (β = 0.52%; *P* < .001), asthma (β = 0.25%; *P* < .001), and stroke (β = 0.15%; *P* < .001) during the last 20 years. The Black-White gap (ie, the change in β between groups) showed improvement (2 trend lines converging) in uninsured status (−0.20%; *P* < .001) and physical inactivity (−0.29%; *P* < .001), while the Black-White gap worsened (2 trend lines diverging) in diabetes (0.14%; *P* < .001), hypertension (0.15%; *P* < .001), coronary heart disease (0.07%; *P* < .001), stroke (0.07%; *P* < .001), and asthma (0.11%; *P* < .001). Hispanic adults showed improvement in physical inactivity (β = −0.28%; *P* = .02) and perceived poor health (β = −0.22%; *P* = .001), while they showed overall deterioration in hypertension (β = 0.79%; *P* < .001) and diabetes (β = 0.50%; *P* < .001). The Hispanic-White gap showed improvement in coronary heart disease (−0.15%; *P* < .001), stroke (−0.04%; *P* < .001), kidney disease (−0.06%; *P* < .001), asthma (−0.06%; *P* = .02), arthritis (−0.26%; *P* < .001), depression (−0.23%; *P* < .001), and physical inactivity (−0.10%; *P* = .001), while the Hispanic-White gap worsened in diabetes (0.15%; *P* < .001), hypertension (0.05%; *P* = .03), and uninsured status (0.09%; *P* < .001).

**Conclusions and Relevance:**

This study suggests that Black-White disparities increased in diabetes, hypertension, and asthma, while Hispanic-White disparities remained in diabetes, hypertension, and uninsured status.

## Key Points

**Question:**

Which health indicators have increased or decreased among Black and Hispanic middle-aged and older adults in the last 20 years?

**Findings:**

In this cross-sectional study of 4 856 326 participants, poor health indicators have consistently trended upward among the Black population, with the highest prevalence ever for most diseases compared with White adults, resulting in increased disparities; for the Hispanic population, disparities in diabetes, hypertension, and uninsured rates are increasing. However, health disparities for physical inactivity in both groups have substantially improved.

**Meaning:**

These findings suggest that continued evidence-based strategies to promote physical activity may be associated with chronic disease management; novel research strategies are necessary to reduce health disparities.

## Teaser

This cross-sectional study evaluates which health indicators have improved or worsened among Black and Hispanic middle-aged and older adults since the Minority Health and Health Disparities Research and Education Act of 2000.

## Introduction

Although racial and ethnic health disparities in the United States have long been recognized as significant issues, these problems persist.^1,2,3,4^ They are rooted in inherited inequality and prejudice, with racial and ethnic minorities experiencing inequity in medical treatment^1,5^ and poor health outcomes.^6,7,8^ In 1999, the 106th US Congress formally asked the Institute of Medicine (currently called the National Academy of Medicine) to systematically assess US health inequities^5^ and draft policy statements on these issues.^9^ A total of $35 billion in federal funds was allocated from 2000 to 2019 to execute 16 461 research projects that attempt to address racial/ethnic disparities, far more than the $12 million spent in the prior 15 years on fewer than 30 projects in this area.^10^

As of June 2020, there were 212 242 PubMed articles relevant to racial/ethnic health disparities. However, peer reviews in health disparities research are vulnerable to political confounders,^2^ and most of these studies report on disparities associated with a few health indicators.^4,8^ To our knowledge, few prior studies have sought to examine the subgroup trends of multiple health indicators simultaneously, by racial/ethnic groups in middle-aged and older adults.^8^

In light of this historical background^1,5,9,11^ and the recent growth in both data sciences^12^ and health disparities research,^6,13^ we sought to examine whether reductions in health disparity indicators^3^ exist across racial/ethnic minority groups since the Minority Health and Health Disparities Research and Education Act of 2000 (passed in 1999).^9^ Focusing on the 2 largest groups of middle-aged^14^ and older racial/ethnic minority groups in the United States, Black (or Non-Hispanic Black or African American [13.4%]) and Hispanic (or Latino [18.3%]),^15^ we compared the 20-year trajectory for multiple health indicators and identified priorities for health disparities–related interventions in this area.^10,16^ Our goal was to provide timely insights to leaders, policy makers, and health professionals^4,5,6,7,8,16^ who can identify priorities for future health equity research.

## Methods

### Design

This repeated cross-sectional study used anonymized, nationally representative data^17^ from the years spanning 1999 to 2018 to answer the research question: “Which health indicators have increased or decreased in disparities among Black and Hispanic middle-aged and older adults in the last 20 years?”^9^ The Columbia University Irving Medical Center institutional review board recognizes that the analysis of deidentified, publicly available data does not constitute human participants research, defined in federal regulations, and is exempt from review; therefore, informed consent was not required. This study followed the Strengthening the Reporting of Observational Studies in Epidemiology (STROBE) reporting guideline.

### Sample

We extracted a total of 4 856 326 records from persons who self-identified as Black (non-Hispanic), Hispanic (non-White), or White and who were 45 years or older in the Behavioral Risk Factor Surveillance System (BRFSS) (January 1999 through December 2018).^17^ Each year, the BRFSS used random dialing of landlines and cell phone numbers, based on a probabilistic sampling strategy, to identify a nationally representative sample of English and Spanish speakers. The randomized probabilistic sampling strategies and criteria used in the BRFSS survey are described in the BRFSS codebooks.^17^ This study targeted adults who were 45 years or older because chronic diseases often emerge in middle age after long-term exposure to harmful behavioral, genetic, environmental, and/or low socioeconomic conditions.^14^ Representation of minority groups in BRFSS data is improving since the 1999 legislation. Our unit of analysis is a prevalence rate (number in 100 persons, expressed as a percentage) with the same denominator for different racial/ethnic groups.

### Variables

Variables from the following survey questions^17^ were used as our poor health indicator variables^2,5,6,7,18^: (1) disease prevalence,^6^ “Have you ever been told by a doctor, nurse, or other health professional that you had [disease name]?” (yes or no for common diseases—diabetes, hypertension, stroke, coronary heart disease [CHD], kidney disease, asthma, chronic obstructive pulmonary disease [COPD], arthritis, and depression); (2) unhealthy behavior,^19^ “During the past month, other than your regular job, did you participate in any physical activities or exercises such as running, calisthenics, golf, gardening, or walking for exercise?” (yes or no; other behaviors such as sleep, diet, alcohol consumption, and smoking were omitted owing to inconsistency of data collection); (3) poor access to health care,^6,20^ “Do you have any kind of health care coverage, including health insurance, prepaid plans such as HMOs (health maintenance organizations), or government plans such as Medicare, or Indian Health Service?” (yes or no); and (4) overall poor health,^21^ “Would you say that in general your health is: 1 = excellent, 2 = very good, 3 = good, 4 = fair, 5 = poor (a response of 4 [fair] or 5 [poor] corresponded to an answer of yes). Our racial and ethnic subgroups were defined by the participants. In this study, all age groups refers to those aged 45 years or older, while middle-aged refers to those in the 45- to 64-year age group.^14^

### Statistical Analysis

Our analyses include 2 parts stratified by age, sex, and race/ethnicity: (1) 20-year trend analyses of poor health indicators^2^ and (2) 10-year prevalence rate comparison between Black and Hispanic adults vs White adults as the reference group.^6,7,18^ We used R, version 3.6.1 (R Foundation for Statistical Computing) and Tableau, version 10.1 (Tableau Software LLC) for our analyses. Trends were applied to project trends forward from 1999 through 2018 in 3 age groups (45-54 years, 55-64 years, and ≥65 years).^22^ An upward slope (β) of 0 or more indicates an increase in the prevalence rate year over year. For prevalence, we included only the most recent 10 years (ie, 2009-2018) because the outcomes of the 1999 legislation would be unlikely to manifest for at least several years after investments in disparities research,^3^ and data on some diseases were collected only since 2010.^17^ Missing rates on outcome variables were less than 1% (mean [SD] missing rates, 0.39% [0.20%]). All *P* values were from 2-tailed tests, and results were deemed statistically significant at *P* < .05. *P* values were adjusted using the Tukey method.^22,23^ We first report on the overall direction of trends, followed by the patterns of disparities (widening or narrowing the gaps).^3^ Slope values are reported as a best-fit yearly change in percentage prevalence (eg, β = 0.5 indicates a 0.5% annual increase in a given health indicator), along with visual displays of 95% CIs among Black and Hispanic adults.^7,18^ Statistical details of trends and prevalence are presented.

## Results

Among the 4 856 326 respondents, the mean (SD) age was 60.4 (11.8) years; 1 285 701 (26.5%) were aged 45 to 54 years, 1 431 568 (29.5%) were aged 55 to 64 years, and 2 139 057 (44.0%) were 65 years or older. A total of 2 958 041 respondents (60.9%) were women; 377 221 (7.8%) self-identified as non-Hispanic Black, 281 951 (5.8%) as Hispanic, and 4 197 154 (86.4%) as non-Hispanic White. A total of 118 043 interviews (2.4%) were conducted in Spanish.

### Black Adults

#### Trends of the Past 20 Years

**Figure 1.**
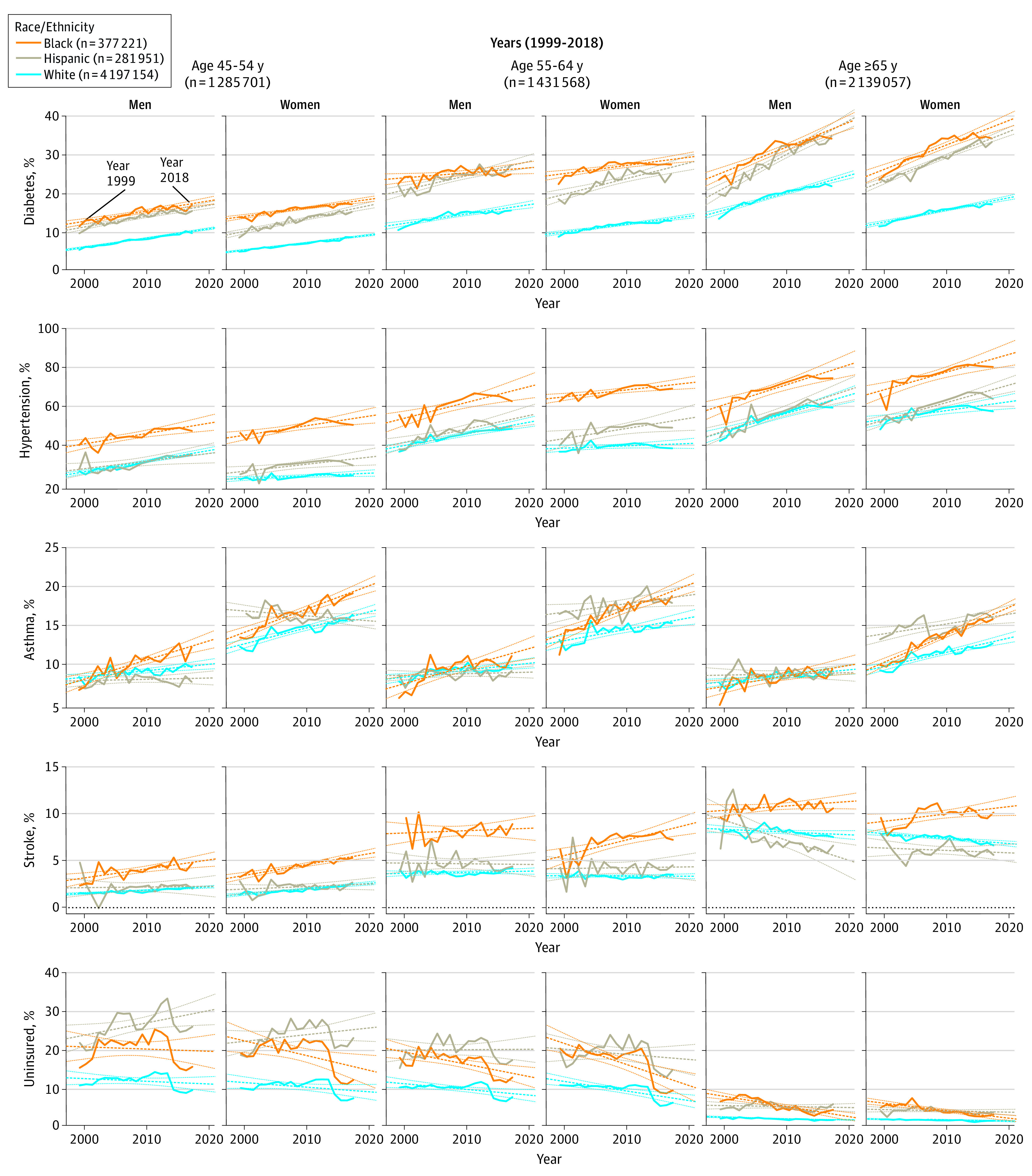
Trends in Health Disparities in the United States During the Past 20 Years (N = 4 856 326)

**Figure 2.**
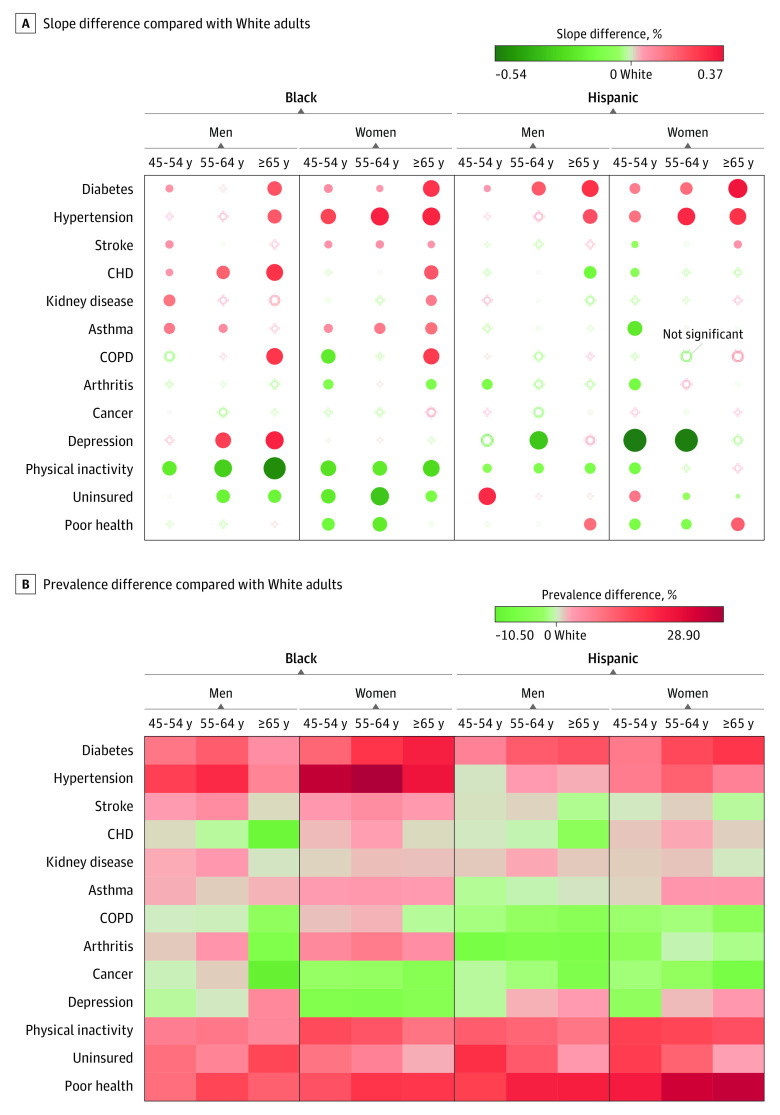
Difference in Slope and the Prevalence of Health Outcomes Compared With White Individuals Among Middle-aged and Older Racial/Ethnic Minority Groups in the US A, Slope difference compared with White individuals, 1999-2018. Red indicates diverging patterns (ie, widening gaps), and green indicates converging pattern (ie, narrowing gaps). The size of the dots corresponds to the magnitude of the slope difference to White adults. B, Prevalence difference compared with White individuals, 2009-2018. Red indicates results that are worse than for Whites adults, while green indicates results that are better than for White adults in terms of prevalence rates; for example, the trend lines for Black and White adults in physical inactivity are in the green light heading toward a right direction, but the prevalence of physical inactivity among Black adults remains unimproved. The β coefficient indicates the slope of yearly percentage of prevalence change. CHD indicates coronary heart disease; COPD, chronic obstructive pulmonary disease.

Overall, Black adults showed a decrease indicating improvement in uninsured status (β = −0.40%; *P* < .001) and physical inactivity (β = −0.29%; *P* < .001), while they showed an increase indicating deterioration in hypertension (β = 0.88%; *P* < .001), diabetes (β = 0.52%; *P* < .001), asthma (β = 0.25%; *P* < .001), and stroke (β = 0.15%; *P* < .001) during the last 20 years (Figure 1 and Table 1). Comparing 2 trend lines between Black and White adults (Figure 2), the Black-White gap (ie, the change in β between groups) showed improvement (2 trend lines converging) in uninsured status (−0.20%; *P* < .001) and physical inactivity (−0.29%; *P* < .001), whereas the Black-White gap worsened (2 trend lines diverging) in diabetes (0.14%; *P* < .001), hypertension (0.15%; *P* < .001), CHD (0.07%; *P* < .001), stroke (0.07%; *P* < .001), and asthma (0.11%; *P* < .001). The trend lines for both cancer and men’s poor general health over time have remained parallel (ie, no significant changes) when comparing Black adults with White adults (Figure 1, Figure 2, and Table 1).

**Table 1.** Trend Analyses of Health Indicators Since US Congress Introduced Legislation to Reduce Racial/Ethnic Health Disparities (N = 4 856 326)

Outcome	Men		Women	
	β (SE)^a^	*P* value^b^	β (SE)^a^	*P* value^b^
**Metabolic disease**				
Diabetes				
Aged 45-54 y				
Black	0.28 (0.03)	<.001	0.23 (0.02)	<.001
Hispanic	0.26 (0.03)	<.001	0.33 (0.03)	<.001
White	0.25 (0.01)	<.001	0.20 (0.01)	<.001
Aged 55-64 y				
Black	0.14 (0.05)	.01	0.21 (0.04)	<.001
Hispanic	0.37 (0.05)	<.001	0.38 (0.06)	<.001
White	0.24 (0.03)	<.001	0.20 (0.02)	<.001
Aged ≥65 y				
Black	0.62 (0.06)	<.001	0.61 (0.06)	<.001
Hispanic	0.83 (0.07)	<.001	0.63 (0.04)	<.001
White	0.44 (0.03)	<.001	0.31 (0.02)	<.001
**Cardiovascular disease**				
Hypertension				
Aged 45-54 y				
Black	0.51 (0.12)	<.001	0.47 (0.12)	<.001
Hispanic	0.38 (0.15)	.03	0.35 (0.13)	.02
White	0.53 (0.04)	<.001	0.13 (0.05)	.02
Aged 55-64 y				
Black	0.79 (0.18)	<.001	0.35 (0.09)	<.001
Hispanic	0.67 (0.15)	<.001	0.79 (0.30)	.02
White	0.58 (0.07)	<.001	0.11 (0.08)	.16
Aged ≥65 y				
Black	0.99 (0.18)	<.001	0.88 (0.18)	<.001
Hispanic	1.02 (0.17)	<.001	0.87 (0.12)	<.001
White	0.91 (0.11)	<.001	0.44 (0.11)	<.001
Stroke				
Aged 45-54 y				
Black	0.11 (0.03)	<.001	0.12 (0.01)	<.001
Hispanic	0.01 (0.04)	.83	0.02 (0.02)	.24
White	0.04 (0.00)	<.001	0.06 (0.01)	<.001
Aged 55-64 y				
Black	−0.03 (0.05)	.62	0.17 (0.03)	<.001
Hispanic	0.00 (0.04)	.91	0.00 (0.05)	.97
White	0.02 (0.01)	.09	0.00 (0.01)	.74
Aged ≥65 y				
Black	0.05 (0.03)	.06	0.10 (0.04)	.04
Hispanic	−0.14 (0.07)	.07	−0.07 (0.11)	.49
White	−0.03 (0.01)	.05	−0.05 (0.01)	<.001
**Respiratory disease**				
Asthma				
Aged 45-54 y				
Black	0.24 (0.03)	<.001	0.30 (0.03)	<.001
Hispanic	0.04 (0.03)	.22	0.06 (0.07)	.36
White	0.09 (0.02)	<.001	0.21 (0.02)	<.001
Aged 55-64 y				
Black	0.22 (0.04)	<.001	0.31 (0.03)	<.001
Hispanic	0.04 (0.03)	.16	0.13 (0.04)	.01
White	0.09 (0.02)	<.001	0.15 (0.03)	<.001
Aged ≥65 y				
Black	0.14 (0.04)	<.001	0.33 (0.02)	<.001
Hispanic	0.03 (0.03)	.42	0.13 (0.04)	<.001
White	0.07 (0.02)	<.001	0.18 (0.02)	<.001
**Behavior**				
Physical inactivity				
Aged 45-54 y				
Black	−0.17 (0.06)	.01	−0.28 (0.06)	<.001
Hispanic	−0.26 (0.10)	.02	−0.28 (0.10)	.01
White	−0.06 (0.09)	.56	−0.08 (0.06)	.23
Aged 55-64 y				
Black	−0.41 (0.08)	<.001	−0.31 (0.05)	<.001
Hispanic	−0.42 (0.10)	<.001	−0.31 (0.11)	.01
White	−0.13 (0.08)	.11	−0.22 (0.06)	<.001
Aged ≥65 y				
Black	−0.52 (0.06)	<.001	−0.43 (0.04)	<.001
Hispanic	−0.30 (0.16)	.08	−0.20 (0.13)	.15
White	−0.24 (0.05)	<.001	−0.34 (0.05)	<.001
**Health care system**				
Uninsured				
Aged 45-54 y				
Black	−0.11 (0.14)	.45	−0.44 (0.14)	<.001
Hispanic	0.30 (0.12)	.03	0.13 (0.12)	.28
White	−0.10 (0.06)	.15	−0.15 (0.07)	.04
Aged 55-64 y				
Black	−0.33 (0.08)	<.001	−0.58 (0.11)	<.001
Hispanic	0.00 (0.11)	1.00	−0.18 (0.13)	.19
White	−0.16 (0.05)	<.001	−0.26 (0.05)	<.001
Aged ≥65 y				
Black	−0.25 (0.04)	<.001	−0.18 (0.03)	<.001
Hispanic	−0.02 (0.03)	.64	−0.02 (0.03)	.37
White	−0.04 (0.01)	<.001	−0.03 (0.01)	<.001

^a^ β Coefficient indicates slope of yearly percentage of prevalence change.

^b^ *P* value for the group difference adjustment: Tukey method for comparing a family of 3 estimates.

#### Recent Prevalence Comparison

When we compared the 10-year prevalence of poor health indicators among Black adults with that among White adults, the prevalence among Black adults was higher for diabetes (10.8%), hypertension (18.6%), uninsured status (7.0%), physical inactivity (8.4%), and perceived poor health (12.3%) (Figure 2 and Table 2). The prevalence among Black adults was lower for cancer (−3.4%), depression (−2.2%), and CHD (−1.0%).

**Table 2.** Recent Prevalence of Health Indicators After US Congress Passed Legislation That Aimed to Reduce Health Disparities (N = 4 856 326)

Disease	Black adults (n = 377 221)				Hispanic adults (n = 281 951)				White adults (n = 4 197 154)			
	1999-2008, %	2009-2018, %	Change, %	*P* value^a^	1999-2008, %	2009-2018, %	Change, %	*P* value^a^	1999-2008, %	2009-2018, %	Change, %	*P* value^a^
**Men aged 45-54 y (n = 528 078)**												
Diabetes	13.8	16.3	2.5	<.001	12.4	14.8	2.4	<.001	6.9	9.0	2.1	<.001
Hypertension	43.4	48.2	4.8	<.001	29.1	34.8	5.7	<.001	29.2	34.6	5.4	<.001
Stroke	3.7	4.6	0.9	<.001	2.1	2.3	0.2	.26	1.7	2.0	0.3	<.001
CHD	3.8	3.9	0.1	.82	4.4	3.5	−0.9	<.001	4.0	3.4	−0.6	<.001
Asthma	10.0	11.8	1.8	<.001	8.8	8.7	−0.1	.79	9.4	9.9	0.5	<.001
COPD	NA	5.3	NA	NA	NA	3.5	NA	NA	NA	5.3	NA	NA
Arthritis	27.7	25.3	−2.4	<.001	20.2	17.0	−3.2	<.001	26.9	24.3	−2.6	<.001
Cancer	NA	2.9	NA	NA	NA	2.2	NA	NA	NA	3.2	NA	NA
Depression	NA	14.6	NA	NA	NA	14.6	NA	NA	NA	15.6	NA	NA
Physical inactivity	30.6	28.8	−1.8	<.001	35.9	32.7	−3.2	<.001	21.8	22.4	0.6	<.001
Uninsured	20.6	19.5	−1.1	.007	25.6	27.5	1.9	<.001	11.8	11.3	−0.5	<.001
Poor health	23.0	23.1	0.1	.68	28.8	28.4	−0.4	.43	13.5	14.7	1.2	<.001
**Women aged 45-54 y (n = 757 623)**												
Diabetes	15.1	16.8	1.7	<.001	11.6	14.4	2.8	<.001	6.0	7.6	1.6	<.001
Hypertension	48.0	52.6	4.6	<.001	30.4	32.7	2.3	<.001	24.3	26.2	1.9	<.001
Stroke	4.0	5.0	1.0	<.001	2.4	2.3	−0.1	.65	1.8	2.2	0.4	<.001
CHD	4.2	3.8	−0.4	.004	4.2	3.4	−0.8	<.001	2.5	2.3	−0.2	<.001
Kidney disease	NA	3.2	NA	NA	NA	3.4	NA	NA	NA	2.5	NA	NA
Asthma	16.3	18.3	2.0	<.001	16.9	16.4	−0.5	.12	14.3	15.7	1.4	<.001
COPD	NA	9.0	NA	NA	NA	5.4	NA	NA	NA	7.7	NA	NA
Arthritis	39.1	35.1	−4.0	<.001	30.6	26.2	−4.4	<.001	33.4	30.2	−3.2	<.001
Cancer	NA	5.4	NA	NA	NA	6.0	NA	NA	NA	8.0	NA	NA
Depression	NA	21.9	NA	NA	NA	24.3	NA	NA	NA	27.7	NA	NA
Physical inactivity	37.0	35.0	−2.0	<.001	39.9	36.4	−3.5	<.001	22.4	22.6	0.2	.10
Uninsured	20.4	17.3	−3.1	<.001	23.5	23.9	0.4	.30	10.7	9.7	−1.0	<.001
Poor health	26.8	27.2	0.4	.29	35.7	34.8	−0.9	.02	14.2	15.7	1.5	<.001
**Men aged 55-64 y (n = 585 081)**												
Diabetes	24.8	25.7	0.9	.05	22.6	25.8	3.2	<.001	13.5	15.2	1.7	<.001
Hypertension	59.2	64.9	5.7	<.001	45.1	50.8	5.7	<.001	43.2	48.1	4.9	<.001
Stroke	8.1	8.4	0.3	.38	4.9	4.6	−0.3	.31	3.6	3.9	0.3	.001
CHD	7.3	7.4	0.1	.86	9.6	7.9	−1.7	<.001	10.1	8.4	−1.7	<.001
Kidney disease	NA	5.9	NA	NA	NA	5.2	NA	NA	NA	3.1	NA	NA
Asthma	9.7	10.9	1.2	<.001	9.2	9.5	0.3	.33	9.6	10.0	0.4	<.001
COPD	NA	8.6	NA	NA	NA	5.8	NA	NA	NA	8.8	NA	NA
Arthritis	41.8	39.7	−2.1	.002	33.0	30.0	−3.0	<.001	39.1	36.3	−2.8	<.001
Cancer	NA	8.3	NA	NA	NA	5.4	NA	NA	NA	7.4	NA	NA
Depression	NA	16.3	NA	NA	NA	18.0	NA	NA	NA	16.2	NA	NA
Physical inactivity	34.4	31.9	−2.5	<.001	39.1	34.1	−5.0	<.001	24.6	24.7	0.1	.45
Uninsured	18.2	14.4	−3.8	<.001	20.3	19.5	−0.8	.09	10.1	8.8	−1.3	<.001
Poor health	32.6	32.7	0.1	.90	40.1	37.7	−2.4	<.001	19.3	19.6	0.3	.05
**Women aged 55-64 y (n = 846 487)**												
Diabetes	26.7	27.8	1.1	.001	22.0	25.0	3.0	<.001	11.0	12.5	1.5	<.001
Hypertension	65.7	69.7	4.0	<.001	47.0	50.6	3.6	<.001	40.0	40.8	0.8	<.001
Stroke	7.2	7.8	0.6	.01	4.3	4.2	−0.1	.78	3.3	3.4	0.1	.64
CHD	7.7	6.8	−0.9	<.001	8.0	6.4	−1.6	<.001	5.3	4.3	−1.0	<.001
Kidney disease	NA	4.8	NA	NA	NA	4.6	NA	NA	NA	3.4	NA	NA
Asthma	16.5	18.4	1.9	<.001	17.7	18.5	0.8	.02	14.5	15.3	0.8	<.001
COPD	NA	12	NA	NA	NA	8.4	NA	NA	NA	10.3	NA	NA
Arthritis	55.8	50.7	−5.1	<.001	48.0	43.6	−4.4	<.001	50.0	44.1	−5.9	<.001
Cancer	NA	8.6	NA	NA	NA	8.3	NA	NA	NA	11.5	NA	NA
Depression	NA	21.0	NA	NA	NA	28.7	NA	NA	NA	27.2	NA	NA
Physical inactivity	39.5	37.1	−2.4	<.001	42.6	38.8	−3.8	<.001	26.6	25.7	−0.9	<.001
Uninsured	18.6	14.1	−4.5	<.001	20.1	17.7	−2.4	<.001	10.2	8.2	−2.0	<.001
Poor health	36.3	34.3	−2.0	<.001	47.4	43.9	−3.5	<.001	19.3	18.7	−0.6	<.001
**Men aged ≥65 y (n = 785 126)**												
Diabetes	29.6	34.0	4.4	<.001	25.4	33.0	7.6	<.001	18.0	21.5	3.5	<.001
Hypertension	66.6	73.8	7.2	<.001	51.2	61.2	10.0	<.001	51.9	59.3	7.4	<.001
Stroke	10.9	11.0	0.1	.77	6.9	6.6	−0.3	.50	8.6	7.9	−0.7	<.001
CHD	9.2	10.4	1.2	.001	15.2	12.8	−2.4	<.001	18.4	16.7	−1.7	<.001
Kidney disease	NA	8.4	NA	NA	NA	6.7	NA	NA	NA	5.7	NA	NA
Asthma	8.6	9.6	1.0	.001	9.3	9.4	0.1	.85	8.9	9.2	0.3	<.001
COPD	NA	9.9	NA	NA	NA	7.6	NA	NA	NA	12.0	NA	NA
Arthritis	49.7	48.1	−1.6	.02	42	39.0	−3.0	<.001	48.2	45.7	−2.5	<.001
Cancer	NA	19.7	NA	NA	NA	12.6	NA	NA	NA	18.8	NA	NA
Depression	NA	10.5	NA	NA	NA	14.0	NA	NA	NA	11.3	NA	NA
Physical inactivity	38.2	33.1	−5.1	<.001	38.3	34.0	−4.3	<.001	27.9	26.7	−1.2	<.001
Uninsured	6.3	3.8	−2.5	<.001	5.5	4.6	−0.9	<.001	1.9	1.5	−0.4	<.001
Poor health	39.0	35.4	–3.6	<.001	45.2	41.1	−4.1	<.001	27.0	22.7	−4.3	<.001
**Women aged ≥65 y (n = 1 353 931)**												
Diabetes	29.8	34.7	4.9	<.001	26.1	31.8	5.7	<.001	14.1	16.6	2.5	<.001
Hypertension	73.6	79.7	6.1	<.001	57.8	65.2	7.4	<.001	55.6	59.4	3.8	<.001
Stroke	10.4	10.1	−0.3	.11	5.9	6.2	0.3	.19	7.7	7.2	−0.5	<.001
CHD	9.5	9.6	0.1	.82	11.8	9.9	−1.9	<.001	10.8	9.1	−1.7	<.001
Kidney disease	NA	6.9	NA	NA	NA	5.7	NA	NA	NA	5.6	NA	NA
Asthma	12.9	15.3	2.4	<.001	14.9	15.9	1.0	.002	11.4	12.6	1.2	<.001
COPD	NA	11.7	NA	NA	NA	8.9	NA	NA	NA	12.8	NA	NA
Arthritis	65.1	62.0	−3.1	<.001	58.6	56.2	−2.4	<.001	60.6	57.7	−2.9	<.001
Cancer	NA	13.0	NA	NA	NA	10.6	NA	NA	NA	18.0	NA	NA
Depression	NA	12.3	NA	NA	NA	20.1	NA	NA	NA	17.3	NA	NA
Physical inactivity	44.3	40.8	−3.5	<.001	48.0	45.1	−2.9	<.001	35.3	33.2	−2.1	<.001
Uninsured	4.8	3.0	−1.8	<.001	4.2	3.5	−0.7	<.001	1.4	1.1	−0.3	<.001
Poor health	42.4	37.4	−5.0	<.001	52.9	48.4	−4.5	<.001	27.2	22.3	−4.9	<.001

Abbreviations: CHD, coronary heart disease; COPD, chronic obstructive pulmonary disease; NA, not applicable.

^a^ By χ^2^ test.

### Hispanic Adults

#### Trends of the Past 20 Years

Overall, Hispanic adults showed improvement in physical inactivity (β = −0.28%; *P* = .02) and perceived poor health (β = −0.22%; *P* = .001), while they showed overall deterioration in hypertension (β = 0.79%; *P* < .001) and diabetes (β = 0.50%; *P* < .001) (Figure 1 and Table 1). Comparing 2 trend lines between Black and White adults (Figure 2), the Hispanic-White gap (ie, the change in β between groups) showed improvement in CHD (−0.15%; *P* < .001), stroke (−0.04%; *P* < .001), kidney disease (−0.06%; *P* < .001), asthma (−0.06%; *P* = .02), arthritis (−0.26%; *P* < .001), depression (−0.23%; *P* < .001), and physical inactivity (−0.10%; *P* = .001), while the Hispanic-White gap worsened in diabetes (0.15%; *P* < .001), hypertension (0.05%; *P* = .03), and uninsured status (0.09%; *P* < .001). The trend lines for kidney disease, COPD, and cancer have remained parallel over time (ie, no significant change) between Hispanic and White adults (Figure 1, Figure 2, and Table 1).

#### Recent Prevalence Comparison

When we compared the 10-year prevalence of poor health indicators among Hispanic adults with that among White adults, the values were higher for Hispanic adults for diabetes (8.9%), hypertension (4.8%), being uninsured (12.7%), physical inactivity (11.7%), and perceived poor health (19.0%) (Figure 2 and Table 2). The prevalence among Hispanic adults was lower for arthritis (−4.4%), cancer (−3.6%), and COPD (−2.9%).

## Discussion

We investigated trends in the population-level prevalence of poor health indicators during the last 20 years.^2,3^ These poor health indicators included chronic diseases, physical inactivity,^6,24^ prevalence of uninsured status,^25^ and overall poor health status^21^ in middle-aged or older Black and Hispanic adults.^9,10^ Despite substantial Congressional funds directed at reducing health disparities during the last 20 years,^10,26^ we found worsened disparities for most diseases among Black adults compared with White adults but fewer disparities among Hispanic adults in comparison with White adults.^8,27^ To our knowledge, this is among the first and largest studies to examine disparities among multidimensional health indicators^6^ over time and with important public policy implications.^2,8^

Overall, Black adults consistently showed the worst trends in physical diseases during the past 2 decades, particularly in diabetes, hypertension, CHD, stroke, kidney disease, and asthma.^4,8,18^ These findings support prior research suggesting that, compared with White adults, Black adults have a higher prevalence of cardiovascular diseases, including hypertension and stroke, as well as kidney diseases. Our findings add to the literature documenting that policy choices have not resulted in the sought-after reductions in Black-White health disparities in the United States.^2,3,7,8,18,28^ Several possible reasons include the fact that the National Academy of Medicine projects were funded primarily to assess the consequences of health disparities and to draft policy statements. Interventions were less emphasized. Initiatives such as the Patient Protection and Affordable Care Act (ACA), an array of policies, were informed by the National Academy of Medicine assessments. The ACA may have led to improvements in disease diagnoses and health care access, including Medicaid and Medicare expansion.^29^ Nonelderly Hispanic adults received the greatest benefit, decreasing the percentage of uninsured individuals by 15%, from 40% to 25%.^30^ Specific to middle-aged and older adults, the ACA includes policies to promote free preventive benefits through Medicare and Medicaid, including screenings for cancer, cardiovascular disease, and diabetes. Several of the chronic diseases that showed the worst trends over time in our data were eligible for screening through ACA policies, suggesting that promoting screening alone will be insufficient to reduce disparities. It may also be the case that there has been no return on investment in these policies and others implemented during the past 20 years and that accurately curving the trajectory of chronic disease will take more time.^3^ It may also be that until social determinants of health (eg, housing, access to food, the local neighborhood and environments, and racism) are addressed, we will continue to see health disparities between Black and White US adults.^4,6,31^ Black and Hispanic Medicare recipients accounted for a disproportionate share of coronavirus disease 2019 (COVID-19) infections and severity.^32,33^ Poor health conditions including diabetes, cardiovascular diseases, kidney disease, asthma, and physical inactivity, as well as uninsured rates among older Black and Hispanic adults (Figure 2), may help us to understand the severity of COVID-19^32^; this warrants future investigation. The ACA increased access to care, yet disparities persist. Such trends should be further captured and examined through BRFSS data collection.

Some poor health trends among Black adults showed improvement. During the past 2 decades, there has been a marked improvement in the Black-White disparity gaps for physical inactivity and uninsured status. A large part of the change in insurance status occurred around 2014 after the passing of the ACA. In contrast to their chronic disease burden, Black adults ranked their general health more favorably than Hispanic adults and showed the healthiest trends in the area of mental health; however, underdiagnosis of mental health conditions among Black adults has been a controversial topic in health care.^34^ Improved physical activity behaviors and access to health care may also explain favorable ratings in self-reported health status and mental health, even in the face of worsening physical health conditions.^35^ According to our analyses, the prevalence of diabetes and hypertension will most likely continue to increase among Black adults from 2020 onward. The potential for self-management of these chronic diseases has implications for the creation of realistic, achievable goals and setting targets for health disparity interventions.^7,8,16,26,36^

Hispanic adults showed complicated patterns in poor health indicators when groups with better and worse outcomes were combined. Relative to Black adults, Hispanic adults showed worsening patterns in diabetes prevalence. In contrast with Black adults, however, Hispanic adults showed lower prevalence in hypertension and stroke, with the lowest prevalence of COPD. This group also showed trend patterns, gradually eliminating the disparities between their White peers in most poor health indicators, including CHD, stroke, kidney disease, asthma, arthritis, depression, and physical inactivity. Future research will be needed to identify evidence-based approaches that are most effective for decreasing disparities.^6,7,29,31,37^

Nonetheless, Hispanic adults ranked worst across all racial/ethnic groups for access to health care during the 20-year period.^6,29^ Despite experiencing a lower chronic disease burden than Black adults, Hispanic men and women of all ages reported the worst perceptions of their general health of all 3 groups.^38^ This finding may be explained by the group’s higher prevalence of mental health challenges, suboptimal insurance coverage, unhealthy behaviors (eg, physical inactivity), or other unmeasured factors (eg, self-management of chronic diseases).^38^ In fact, Hispanic men aged 45 to 54 years ranked worst for physical inactivity, with rates similar to those among White women aged 65 years or older. Hispanic individuals’ poor perception of their general health may also be associated with language; a correlation was observed between a BRFSS Spanish interview^39^ and the increased likelihood to report poor health (eg, poor general health, diabetes, and physical inactivity).^38^ Future research should explore the intersection of Hispanic ethnicity and acculturation-related factors that are associated with disparity trends over time and underdiagnosed diseases.^40,41^ Our findings also support the need for culturally sensitive social support in the realms of mental health care and promoting physical activity.^6,7,8,36^

During the past 20 years, the Minority Health and Health Disparities Research and Education Act of 2000^9^ resulted in the allocation of $35 billion in federal funds for the implementation of 16 461 projects addressing racial/ethnic disparities.^10,16^ Despite substantive national progress toward health equity, our study confirms that health disparities persist for outcomes associated with the most common diseases among Black adults and in diabetes and hypertension among Hispanic adults. To ensure improved outcomes in 2030, we must overcome inertia and resolve tensions surrounding these issues.^2,3,6,8^ Other national initiatives exist to address disparities, including the Department of Health and Human Services (HHS): Healthy People Goals, HHS Million Hearts, and the HHS Disparities Action Plan. As seen in each 10-year assessment of the Healthy People Goals, progress can be challenging and slow. We encourage researchers to look to the successes thus far in the areas of physical inactivity and arthritis, which will allow us to advance our national health equity mission.^7,9,18^ Researchers and policy makers should continue to propose targeted, culturally sensitive, multilevel interventions that can be widely implemented and sustained.^2,3,6,7,8,36^

### Limitations

This study has some limitations. The validity of our findings in this observational study is limited by the nature of using telephone-based and self-reported cross-sectional survey data. Furthermore, this study provides an incomplete 20-year picture of racial/ethnic health care disparities.^3,5,6,9^ For example, rates of undiagnosed diabetes are estimated to be 20% to 40%, which may be higher among Hispanic adults, considering uninsured rates.^40,41^ Although the BRFSS has continuously improved in sampling strategies over time for racial/ethnic minority groups and has the world’s largest amount of survey data, our results might have inherited the limitations of using underrepresented minority samples in earlier years since the 1999 legislation. Although significant public funding was allocated to reduce disparities in HIV/AIDS,^10^ we were unable to report on HIV/AIDS^2,18^ because of the unavailability of these BRFSS data. In addition, we were able to document only 1 behavior^21^ using a single self-report item. Furthermore, separate prevalence estimates were not conducted for linguistically unacculturated immigrants vs US-born respondents across racial/ethnic groups.

## Conclusions

In the 20 years since the Minority Health and Health Disparities Research and Education Act of 2000 was written into law, we found evidence of both diverging and converging health trends among Black and Hispanic adults vs White adults in the United States. Racial/ethnic disparities continue to persist in diabetes, hypertension, CHD, and asthma among Black adults. Racial/ethnic disparities persist in diabetes, hypertension, mental health, and uninsured status among Hispanic adults. Nevertheless, our trend analyses found a clear narrowing of health disparities in both physical inactivity and arthritis in both Black and Hispanic adults compared with White adults. The prevalence of uninsured status among Black and Hispanic adults compared with White adults has decreased in the past 20 years, with the greatest reduction in disparities in insurance coverage seen among Hispanic adults.
